# Experimental Performance Analysis of Large-Format 304 Stainless Steel Surface Laser Matting Process

**DOI:** 10.3390/ma18184412

**Published:** 2025-09-22

**Authors:** Qianqian Ding, Mingdi Wang, Xihuai Wang, Peijiao Huang, Zirui Wang, Yeyi Ji

**Affiliations:** 1 School of Precision Manufacturing, Suzhou Vocational Institute of Industrial Technology, Suzhou 215104, China; dingqian0922@126.com (Q.D.);; 2 School of Mechanical and Electrical Engineering, Soochow University, Suzhou 215000, China; 3 College of Information Technology, Mechanical Engineering and Automotive Transport, T. F. Gorbachev Kuzbass State Technical University, Kemerovo 650000, Russia

**Keywords:** large format, 304 stainless steel, laser matting processing, process parameters, switching light delay

## Abstract

In response to the demand for rapid matte finishing on large-format 304 stainless steel surfaces, this study utilized four fiber laser devices (output wavelength: 1064 nm, output power: 100 W, maximum modulation frequency: 4 kHz) to simultaneously perform surface matte finishing experiments on 304 stainless steel, with the aim of fabricating anti-reflective micro-nano structures. During the experiments, by systematically investigating the influence of parameters—including laser power, scanning speed, frequency, and idle speed of a single laser head—on the matte finishing process, the optimal processing parameters for a single laser head were determined as follows: laser power of 20 W, scanning speed of 11,000 mm/s, and frequency of 80 kHz. For large-area high-speed laser matte finishing, the delay of laser on/off was adjusted to compensate for the galvanometer’s motion trajectory, thereby ensuring uniform ablation at both the start and end positions of the processing path. Furthermore, in the context of large-area rapid multi-head laser matte finishing on 304 stainless steel, the overlapping of surface regions processed by different galvanometers was achieved by calibrating the motion start and end points of each galvanometer. The optimal overlapping parameters were successfully obtained. This study provides technical support for environmentally friendly matte finishing of stainless steel and offers valuable insights for its application in the stainless steel home appliance industry.

## 1. Introduction

304 stainless steel is widely used in the food industry, medical devices, furniture, and other fields owing to its high gloss, excellent corrosion resistance, and good processability. With the continuous improvement of quality of life, there are growing demands for enhanced esthetic appeal of stainless steel surfaces, among which color and matte treatments have become important research directions [1,2]. Current matte finishing techniques for stainless steel include sandblasting, chemical etching, and electrochemical polishing. However, sandblasting generates airborne dust, and prolonged exposure may lead to severe respiratory diseases such as pneumoconiosis. Chemical etching and electrochemical polishing processes involve the use of corrosive solutions containing heavy metal ions, resulting in serious environmental pollution [3,4,5]. In the context of increasing emphasis on green manufacturing, the industrial sector has imposed stricter requirements for environmental protection and sustainable production. As a result, there is an urgent need to develop a novel, non-polluting matte finishing method for stainless steel.

Laser matte finishing, as a subset of laser-based metal surface coloration technology, is a non-contact processing method. Since it does not physically contact the material surface during processing, it effectively avoids adverse effects induced by mechanical stress [6,7]. This technique allows high-precision treatment of stainless steel surfaces and significantly reduces environmental pollution associated with conventional processing techniques. As a result, it is recognized as an emerging green and environmentally friendly manufacturing technology.

Currently, scholars both domestically and internationally have conducted extensive research on laser processing of materials using femtosecond and nanosecond lasers. In the field of laser cutting defect analysis, RAJESH et al. [8] investigated the characteristics of CO_2_ laser cutting of stainless steel. Their findings indicate that appropriate laser power and cutting speed can achieve high cutting quality, whereas improper parameter selection may result in uneven cut surfaces or an excessively large heat-affected zone. Sharma et al. [9] studied material transfer and surface properties during fiber laser cutting of galvanized steel sheets. They demonstrated that laser power and cutting speed significantly influence cut quality and material transfer behavior, with deposits formed during the process also affecting surface integrity. Irene et al. [10] developed a regression model based on multifactorial experiments to analyze the effects of pulse frequency, pulse width, and cutting speed on the surface roughness, burr thickness, and dimensional accuracy of stainless steel plates. Their results show that pulse frequency has the greatest influence on roughness and burr formation, while pulse width is the dominant factor affecting dimensional error. In the field of laser color processing, Anatoliy Y. Vorobyev [11,12] used high-repetition-rate femtosecond laser pulses for direct writing to etch multifunctional surfaces, such as ultra-wideband absorption, super-hydrophobic, and self-cleaning surfaces. Wu Yonghua et al. [13] used femtosecond lasers to process and induce different colors on the stainless-steel surface, achieving research on the coloring effects of various laser-processing parameters on different colors. Peixun Fan et al. [14,15] adopted a direct-writing strategy of pulse-injection-controlled ultrafast lasers and used two different processing methods for composite processing (a hybrid structure with a large number of nanoparticles attached to regularly arranged micro-cones). In the ultraviolet to near-infrared wavelength range, the average reflectivity of titanium alloy reached about 2.4%. Based on the above research results, the large-format and rapid laser extinction technology for stainless-steel surfaces has clear engineering significance for the practical application and promotion of laser-based extinction treatment on stainless-steel surfaces [16].

In this study, a laser matte finishing system was employed to process the surface of stainless steel materials. Different sets of process parameters were applied to treat 304 stainless steel samples. Through comparative experiments under various conditions, the optimal parameter combination for surface matte finishing was determined using orthogonal experimental analysis. Subsequently, trajectory compensation through laser on/off delay control was implemented to achieve uniform spot size and consistent ablation at the junctions between processing regions of different galvanometers. High-quality seamless splicing at the processing boundaries was further realized by calibrating the XY-direction offset among the four galvanometers. Finally, rapid matte finishing over large-format stainless steel surfaces was accomplished via synchronized multi-galvanometer processing.

## 2. Materials and Methods

### 2.1. Materials

In this experiment, 304 stainless steel produced by Shanghai Teddy Trading Co., Ltd. (Shanghai, China) was used, with dimensions of 120 cm × 60 cm and a thickness of 1.5 mm. Its chemical composition is shown in Table 1 below. Before carrying out the laser matte treatment, it is first necessary to pretreat the surface of the 304 stainless steel sample, that is, to polish the surface of the stainless steel, then clean it with absolute ethanol and dry it for later use.

### 2.2. Experimental Equipment

In this experiment, a self-designed three-dimensional motion platform for laser-based matte finishing of stainless steel surfaces was employed. The system primarily consists of a servo motor-driven 3D motion platform and laser processing galvanometer units. A gantry-type layout was adopted in this study, enabling simultaneous processing with four laser optical heads. The pulsed laser source used was a YDFLP-E2-100-M7 (JPT Opto-electronics Co., Ltd., Shenzhen, China), with an output wavelength of 1064 nm, a maximum power of 100 W. The key optical component, the galvanometer, was a 10D two-dimensional model produced by Zhibotaike Co., Ltd. (Shenzhen, China), featuring a 10 μm spot size and designed specifically for high-speed flying laser processing.

**Figure 1:**
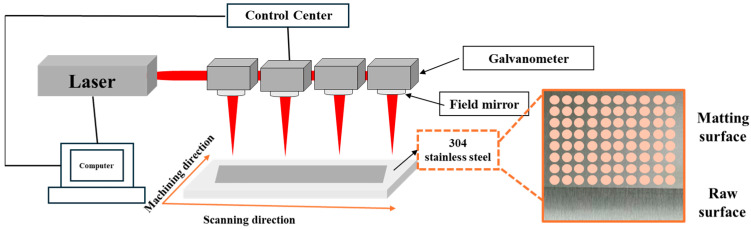
Schematic of laser matting treatment of stainless steel surface.

During the laser matte finishing process on stainless steel, a linear laser spot was utilized. The scanning trajectory of the spot was jointly controlled by the feed mechanism and the galvanometer. The laser beam was deflected by the galvanometer into the processing field and focused onto the surface of the 304 stainless steel workpiece. The movement of the laser spot, achieved through galvanometer-driven scanning, enabled multi-directional processing of the material. A schematic diagram of the processing principle is provided in Figure 1. The spot scanning speed was adjusted by regulating the rotational speed of the galvanometer, while the feed rate was controlled precisely by the servo motor.

### 2.3. Laser Matting Path

**Figure 2:**
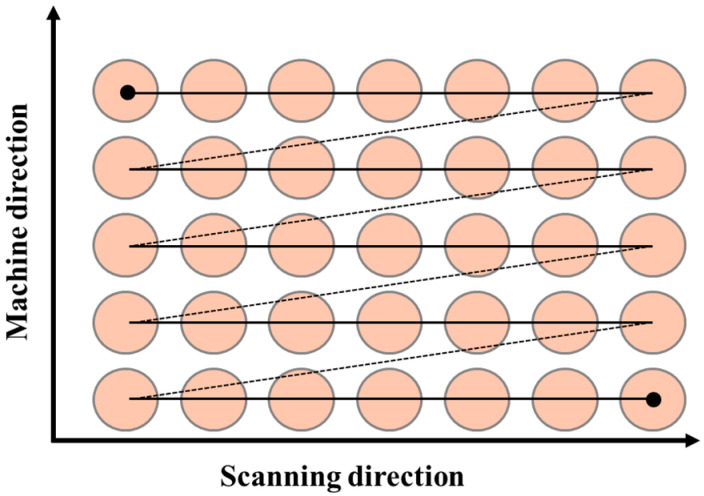
Scanning routes of laser spot.

In the single optical head laser processing experiment, this paper selects laser power, galvanometer scanning speed, laser frequency, and idle speed as the process parameters to be studied, and further explores the relationship between different laser parameters and the quality inspection of laser-processed surfaces. On the premise of ensuring processing quality, this study adopts a unidirectional raster scanning mode with a spacing of 0.15 mm, and uses a pattern size of 140 mm × 13.8 mm and a unidirectional raster scanning mode with a spacing of 0.15 mm, as shown in Figure 2 [17].

### 2.4. Detection Equipment

The 304 stainless steel surface of each sample was determined using a GP 2010-40K optical microscope (Kunshan Gaopin Precision Instrument Co., Ltd., Suzhou, Jiangsu, China) at 100× magnification. The sessile drop method was used to test the surface contact angle of the cleaned area. The contact angle measuring instrument used was Shengding Precision SDC-200S (Shengding Precision Co., Ltd., Dongguan, China), with a contact angle measurement range of 0°~180° and a droplet volume of 5 μL. The average value after three measurements was taken as the recorded result. The glossiness was measured using a YG268 three-angle high-precision gloss meter (3nh Technology Co., Ltd., Guangzhou, Guangdong, China).

## 3. Results and Discussion

### 3.1. Analysis of Single-Beam Laser Processing Technology

The parameters involved in the laser matte finishing process are influenced by multiple factors and levels, each differentially affecting the degree of surface matting on stainless steel. To evaluate the significance of each factor and identify the optimal parameter combination for matte finishing, the processing quality was characterized using two indicators: contact angle and glossiness. This approach enables the determination of the best processing parameters for achieving high-quality large-format laser matte effects and provides a foundation for subsequent painting treatments [18,19,20]. This study focuses primarily on key laser process parameters, namely laser power, galvanometer scanning speed, and scanning frequency. To ensure experimental efficiency, four levels were selected for each factor [21,22,23,24]. Based on preliminary single-factor experiments, the parameter ranges for the orthogonal experimental design of laser matte finishing were established, as detailed in Table 2.

**Figure 3:**
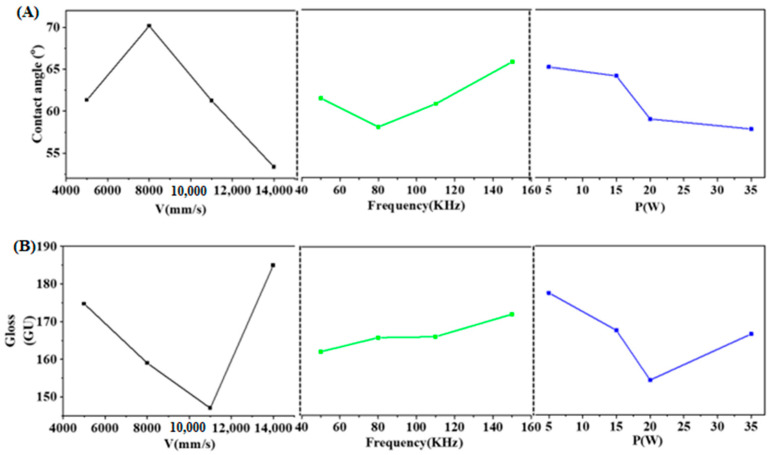
Experimental results under different process parameters: (**A**) Contact angle; (**B**) Gloss.

**Figure 4:**
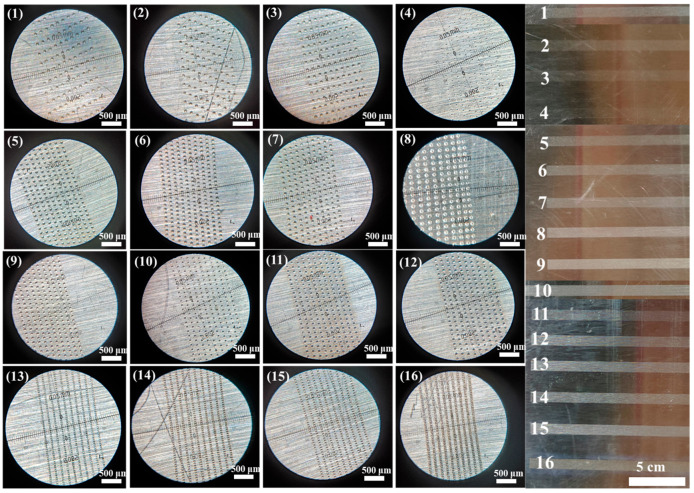
Microscopic and macroscopic morphology of orthogonal experimental samples.

The three factors in this experiment, galvanometer scanning speed, frequency and power are all four level factors, according to the L_16_ (4^3^) orthogonal experiment table to design the orthogonal experiment, the gloss and hydrophilicity after laser matte processing are an important factor to test the laser matte treatment standard, so the contact angle and gloss are used as the detection standards of laser matte treatment, which is used to optimize the experimental parameters as shown in Table 3, and the results are designed as shown in Table 4 by the extreme difference analysis of the indexes. The Contact angle and gloss experimental results under different process parameters are shown in Figure 3. The surface morphology of the sample observed by light microscopy is shown in Figure 4 [25].

The results of the orthogonal experiments are presented in Figure 4. As shown in the figure, when the laser energy density is relatively low, the interaction between the laser and the stainless steel surface typically produces fine pits or granular structures. With further increase in energy density, the incident laser induces significant ablation on the surface, forming localized molten pools that result in thermal melting and material spattering. As the laser energy is conducted and absorbed by the material in the form of heat, the stainless steel surface undergoes rapid cooling and solidification, leading to the formation of micro-cavity structures distributed in an interlaced pattern [26].

Due to the minimal correlation between the two indicators of contact angle and gloss on the surface of the samples after laser matte processing, their patterns of change differ with variations in process parameters. Consequently, it is not possible to achieve optimal processing effects simultaneously for both indicators; thus, it is necessary to conduct experiments focusing on either indicator to obtain the optimal process parameters that allow both to reach their best states, specifically achieving a contact angle of less than 60° on the surface of stainless steel post-laser matte treatment. According to the range analysis results in Table 4, we can determine the relationship between the laser processing parameters and the contact angle. In our analysis, we chose to conduct range analysis only for the contact angle, obtaining its range value R. It can be observed that the order of influence among the three factors is: V > f > P, indicating that the impact of the scanning speed of the galvanometer holds the most weight, while the effect of power is the least significant. Since subsequent treatment of the stainless steel surface involves painting, a smaller contact angle is preferable to enhance its affinity; therefore, the optimal process parameters for the contact angle indicator yield: V1f3P2. We then analyzed the range value for the glossiness indicator and discovered that the order of influence factors is: V > P > f, meaning that the scanning speed of the galvanometer has the greatest impact on glossiness, while the scanning frequency has the least effect. Figure 3 shows the influence trends of the three laser factors—galvanometer scanning speed, frequency, and power—on the evaluation indexes. Among them, Figure 3A presents the contact angle index, and Figure 3B presents the glossiness index. From the trend graphs, it can be confirmed that the galvanometer scanning speed has the greatest influence on the contact angle and glossiness. Therefore, when determining the optimal process parameters, the galvanometer scanning speed should be the primary focus [27,28]. Meanwhile, the analysis of the process results, derived from the micrographs and optical photographs in Figure 4, is as follows:

1. Galvanometer scanning speed (V): The scanning speed is the primary influencing factor for both contact angle and glossiness. Matte finishing can be achieved on stainless steel at scanning speeds of 14,000 mm/s and 11,000 mm/s. However, at 14,000 mm/s, the contact angle is comparatively lower and the matte appearance is less uniform; therefore, a scanning speed of 11,000 mm/s is preferred.
2. Frequency (f): Laser frequency is the second most significant factor affecting contact angle and the third for glossiness. Both 50 kHz and 80 kHz are feasible for matte processing; however, a frequency of 80 kHz results in a smaller contact angle and is thus selected as the optimal value.
3. Power (P): Laser power ranks third in influencing glossiness and second in affecting contact angle. The optimal power for stainless steel matte finishing is determined to be 20 W.

**Figure 5:**
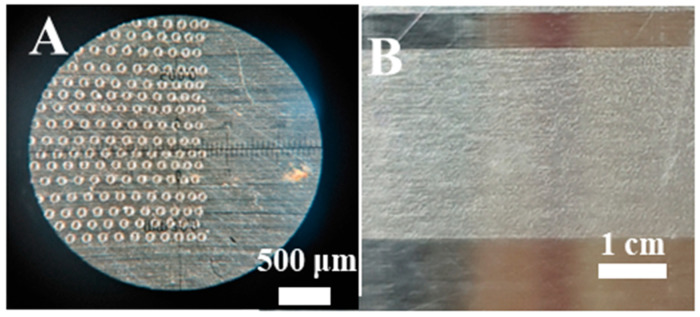
(**A**) Microscopic morphology and (**B**) macroscopic morphology of matte stainless steel surface.

Based on the analysis of the experimental data mentioned above, in the subsequent rapid ablation experiments with multiple galvanometers for large-format lasers, the processing parameters selected for each galvanometer were a scanning speed of 11,000 mm/s, a frequency of 80 kHz, and a power of 20 W. The microstructure of the treated 304 stainless steel surface is shown in Figure 5A, while the macroscopic surface is shown in Figure 5B. The surface glossiness measured 149 GU, meeting the requirements for applications in the stainless steel home appliance industry.

### 3.2. Research on the Laser Dulling of Stainless Steel Surfaces of Four Laser Processing Equipment

#### 3.2.1. Trajectory Compensation for the Switch Light Delay

**Figure 6:**
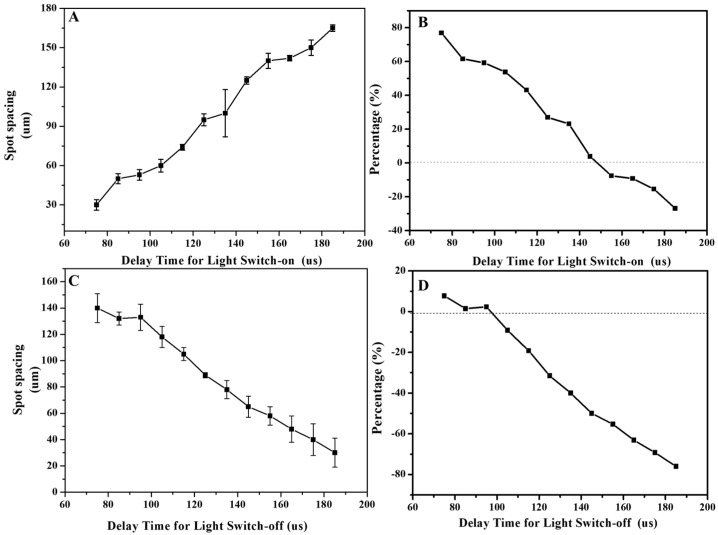
(**A**) The relationship between the opening delay and the spot spacing; (**B**) Corresponding spacing error plot of the opening delay; (**C**) The relationship between the light off delay and the spot spacing; (**D**) Corresponding spacing error plot of the light off.

Utilizing the optical head of the four galvanometers to complete the matte processing of large-format stainless steel, the most important experimental aspect is the splicing of the four processing surfaces of the galvanometers, ensuring the consistency of the matte finish on the stainless steel surface. Due to the mechanical movement of the reflective lens in the laser scanning galvanometer device, the deflection dynamics of the beam cannot be fully synchronized with the opening or closing state of the laser when the galvanometer is scanning in one direction, and the processing uniformity of the processing start and end positions will occur, so we need to adjust the trajectory compensation of the switching optical delay before splicing [29]. The delay for turning the laser on and off should be neither too long nor too short. When the laser-on delay is too short, the galvanometer is either not accelerated or in the acceleration phase, with its actual speed lower than the theoretical speed. Compared to the uniform speed region, the starting position accumulates laser energy for a longer time, resulting in a darker spot (or “over-irradiated spot”) after processing. When the laser-on delay is too long, the galvanometer has already finished accelerating and entered the uniform motion phase. Although the energy accumulation is uniform in this case, a section of the trajectory is unprocessed, making the actual length shorter than the theoretical length. Similarly, when the last coordinate is input to the galvanometer, the laser will wait for a period specified by the laser-off delay parameter before turning off. It can be seen that if the laser-off delay is too short, the galvanometer has not yet started decelerating, leading to a shorter trajectory length. If the laser-off delay is too long, the galvanometer will start decelerating or have reached the final coordinate, resulting in a darker spot in the end region of the trajectory. Subsequently, we carried out the relationship between the consecration and off-light delay and the spot spacing, and the spot spacing and spacing error obtained between 75 us and 185 us were regulated for different consecration delays, as shown in Figure 6A. Figure 6B is the corresponding spacing error plot of the opening delay. The spot spacing and spacing error obtained between 75 us and 185 us are shown in Figure 6C. Figure 6D is the corresponding spacing error plot of the light off. It can be seen from the figure that under the optimal process parameters, the standard spot spacing is 0.13 mm, the spot spacing error is ≤20%, and the corresponding optimal switching optical delay is 145 us and 95 us, respectively.

#### 3.2.2. Research on the Offset of the Four Galvanometer Centers Relative to Galvanometer 1

**Figure 7:**
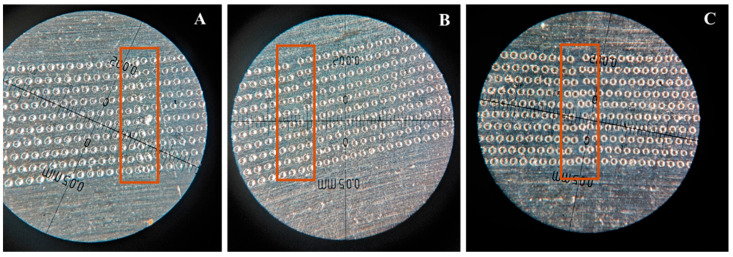
Partial enlargement of the splicing of galvanometers (**A**) 1–2; (**B**) 2–3; and (**C**) 3–4.

Employing the aforementioned optimized process parameters, large-area high-speed laser matte finishing was performed on the stainless steel surface. During the experiment, four small-field galvanometers were utilized for simultaneous processing. A critical challenge encountered in this multi-head operation was the seamless splicing of regions processed by different galvanometers, which necessitated precise alignment of the laser processing origins. To address stitching inaccuracies, software-based adjustments were applied to calibrate the positional offsets of Galvanometers 2, 3, and 4 relative to Galvanometer 1 in both the X and Y directions. These compensations effectively minimized visible seams between adjacent processing zones, enabling uniform large-area matte finishing. The specific offset values derived from experimental calibration are summarized in Table 5. An overview of the stitching result is provided in Figure 6, and the corresponding macroscopic appearance is shown in Figure 7.

### 3.3. Practical Applications of Industrialization in Production

**Figure 8:**
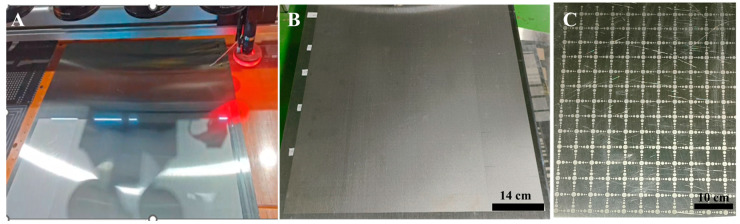
Macro images of large-format laser matte-treated stainless steel (**A**) Processing diagram; (**B**) Large-format matte image; (**C**) Large-format pattern.

Based on the optimized process parameters described above, the operating software was configured with the following settings: galvanometer scanning speed of 11,000 mm/s, laser power of 20 W, frequency of 80 kHz, and light-on/light-off delays of 145 μs and 95 μs, along with relative offset values for the centers of Galvanometers 2–4 with respect to Galvanometer 1. Laser processing was subsequently carried out on the stainless steel surface. A representative image of the processing procedure is provided in Figure 8A. The macroscopic view of the treated surface (Figure 8B) shows no visible seams and exhibits a uniform gloss level of 149 GU. Under practical production conditions, at the scanning speed of 11,000 mm/s, the measured processing speed reached 0.96 cm/s (34.56 m/h). With all four optical units operating simultaneously over a processing width of 63.6 cm, an effective processing area of 21.98 m^2^/h was achieved. These results indicate that the processing efficiency is competitive with alternative methods, while also representing a green, pollution-free, and environmentally friendly manufacturing approach. Furthermore, Figure 8C demonstrates successful pattern printing on the stainless steel surface, confirming the feasibility and applicability of the laser matte finishing process for stainless steel.

## 4. Conclusions

This study systematically investigates three critical aspects of laser matte finishing: the process parameters for single-head laser processing, the delay patterns of galvanometer control, and the center-offset calibration for multi-galvanometer systems. The objective is to establish an optimized experimental process and provide key technologies for the industrial-scale high-speed matte finishing of large-format 304 stainless steel. The main conclusions are as follows:

1. In the single-head laser matte finishing experiments, orthogonal experiments were designed considering laser power, frequency, and scanning speed. Range analysis of the experimental results indicates that the galvanometer scanning speed has the most significant influence on the contact angle, while laser power exhibits the least effect on glossiness. The optimal process parameters for single-galvanometer processing are determined as follows: laser power of 20 W, scanning speed of 11,000 mm/s, and frequency of 80 kHz.
2. Large-format 304 stainless steel was processed using a four-galvanometer simultaneous operation strategy. To address the challenge of seamless stitching between processing zones, laser distance compensation was first achieved by calibrating the light-on delay of the galvanometers. Under the optimal single-head parameters, where the standard spot pitch is 0.13 mm, the optimal light-on delays were identified as 145 μs and 95 μs. Furthermore, the center offsets of the additional galvanometers relative to Galvanometer 1 were precisely adjusted: in the X-direction, the offsets of Galvanometers 2, 3, and 4 were 156.1 mm, 315 mm, and 469.49 mm, respectively; in the Y-direction, the corresponding offsets were 1.3 mm, 1.58 mm, and 1.25 mm. This configuration enabled uniform and efficient matte finishing over large areas, achieving high-quality processing without visible seams.

## Figures and Tables

**Table 1:** Chemical composition of 304 stainless steel.

Element	C	N	Si	P	S	Cr	Mn	Fe	Ni
mass %	0.056	0.0755	0.366	0.022	0.008	19.1	1.17	bal.	8.65

Bal. represents balance.

**Table 2:** Factor level table.

Level	Factor		
	Scanning Speed V [mm/s]	Frequency F [kHz]	Power P [W]
1	14,000	150	35
2	11,000	110	20
3	8000	80	15
4	5000	50	5

**Table 3:** Orthogonal test element arrangement and results.

Number	Scanning Speed [mm/s]	Frequency [kHz]	Power [W]	Contact Angle [°]	Gloss [GU]
1	14,000	150	35	56.7	185
2	14,000	110	20	62.4	165
3	14,000	80	15	43.7	189
4	14,000	50	5	50.6	201
5	11,000	150	20	57.9	142
6	11,000	110	35	46.8	158
7	11,000	80	5	68.3	152
8	11,000	50	15	72	136
9	8000	150	15	80.6	169
10	8000	110	5	73.8	165
11	8000	80	35	65.4	157
12	8000	50	20	60.9	145
13	5000	150	5	68	192
14	5000	110	15	60.2	176
15	5000	80	20	54.8	165
16	5000	50	35	62.3	166

**Table 4:** Range analysis results.

	Number	Scanning Speed	Frequency	Power
	K1	53.35	65.8	61.675
	K2	61.25	60.8	59
Contact angle	K3	70.175	58.05	64.125
	K4	61.325	61.45	63.4
	R	16.825	7.75	5.125
	Order of the factors V > f > P			
	Optimal parameter set V1f3P2			
	K1	185	172	168.5
Gloss	K2	147	166	154.25
	K3	169	168.75	167.5
	K4	174.75	162	177.5
	R	38	16	23.25
	Order of the factors V > P > f			
	Optimal parameter set V2P2f4			

**Table 5:** Offset values of the center of the quadrupole for galvanometer 1.

Offset to the Center of Galvanometer 1	Galvanometer 2 X [mm]	Galvanometer 2 Y [mm]	Galvanometer 3 X [mm]	Galvanometer 3 Y [mm]	Galvanometer 4 X [mm]	Galvanometer 4 Y [mm]
Value	156.1	1.3	315	1.58	469.49	1.25

## Data Availability

The original contributions presented in this study are included in the article. Further inquiries can be directed to the corresponding authors.
